# A Systematic Assessment of Smartphone Tools for Suicide Prevention

**DOI:** 10.1371/journal.pone.0152285

**Published:** 2016-04-13

**Authors:** Mark Erik Larsen, Jennifer Nicholas, Helen Christensen

**Affiliations:** 1 Black Dog Institute, University of New South Wales, Sydney, New South Wales, Australia; 2 School of Psychiatry, Faculty of Medicine, University of New South Wales, Sydney, New South Wales, Australia; University of Stellenbosch, SOUTH AFRICA

## Abstract

**Background:**

Suicide is a leading cause of death globally, and there has been a rapid growth in the use of new technologies such as mobile health applications (apps) to help identify and support those at risk. However, it is not known whether these apps are evidence-based, or indeed contain potentially harmful content. This review examines the concordance of features in publicly available apps with current scientific evidence of effective suicide prevention strategies.

**Methods:**

Apps referring to suicide or deliberate self-harm (DSH) were identified on the Android and iOS app stores. Systematic review methodology was employed to screen and review app content. App features were labelled using a coding scheme that reflected the broad range of evidence-based medical and population-based suicide prevention interventions. Best-practice for suicide prevention was based upon a World Health Organization report and supplemented by other reviews of the literature.

**Results:**

One hundred and twenty-three apps referring to suicide were identified and downloaded for full review, 49 of which were found to contain at least one interactive suicide prevention feature. Most apps focused on obtaining support from friends and family (n = 27) and safety planning (n = 14). Of the different suicide prevention strategies contained within the apps, the strongest evidence in the literature was found for facilitating access to crisis support (n = 13). All reviewed apps contained at least one strategy that was broadly consistent with the evidence base or best-practice guidelines. Apps tended to focus on a single suicide prevention strategy (mean = 1.1), although safety plan apps provided the opportunity to provide a greater number of techniques (mean = 3.9). Potentially harmful content, such as listing lethal access to means or encouraging risky behaviour in a crisis, was also identified.

**Discussion:**

Many suicide prevention apps are available, some of which provide elements of best practice, but none that provide comprehensive evidence-based support. Apps with potentially harmful content were also identified. Despite the number of apps available, and their varied purposes, there is a clear need to develop useful, pragmatic, and multifaceted mobile resources for this population. Clinicians should be wary in recommending apps, especially as potentially harmful content can be presented as helpful. Currently safety plan apps are the most comprehensive and evidence-informed, for example, “Safety Net” and “MoodTools—Depression Aid”.

## Introduction

### Rationale

Suicide is a leading cause of death globally, particularly amongst young people [1]. Although immediate help during a crisis is critical, those who may be experiencing suicidal ideation or crisis experience barriers to help-seeking, such as not perceiving a need for professional help, lack of time, preference for informal help, access to and cost of services, and fear of stigma and disclosure [2]. With the increasing ubiquity of mobile phones, health applications (apps) have the potential to improve access and availability of evidence-based support to this group, as apps are low-cost, convenient, and discreet. Apps may be especially suited to deliver suicide prevention interventions with their ability to deliver support and intervention in situ and at the time of crisis. As suicide ideation and suicide risk change rapidly, access to high quality mobile resources may save lives.

Consumers are rapidly embracing apps, proactively seeking apps to manage their personal health. Recent data suggest 85% of young people in the USA own a smartphone, three quarters of whom have used their device to access health information [3]. In a survey in the psychiatric out-patient setting, 69% of respondents and 80% of those aged 45 years or younger indicated a desire to use a mobile app to track their mental health [4].

This consumer enthusiasm for apps to manage mental health has spurred the development of numerous apps for suicide prevention. Many of these offer digitised versions of tools and strategies common in mental health. However, to our knowledge the content of these apps has not been investigated. There is also an absence of efficacy data for apps related to suicide prevention, although the publication of designs [5], proof-of-concept results [6], and protocols for evaluation studies [7] are indicative of the future research direction. Assessment of content is vital, as the Android [8] and iOS [9] app stores do not have guidelines specifically related to the restriction of pro-suicidal content, or app content quality. Therefore, we currently do not know whether apps provide potential harmful content which promotes suicide or encourages suicidal behaviour [10], nor whether their content is consistent with clinical and population based policy.

In previous work, Donker *et al*. found that mental health apps evaluated in randomised controlled trials [11] were not publicly available, while those with no research evidence were. Reviews of apps for other mental and physical disorders support this, reporting low adherence to clinical best practice, or the provision of unreliable, unsuitable tools [12–14]. In the present review, in view of the absence of published efficacy data for existing apps, we use the corpus of extant research trial evidence to address whether the features of publicly available health apps for suicide prevention are consistent with the research evidence. Suicide prevention strategies identified within the apps were ranked according to the strength of the evidence, as indicated by inclusion in the World Health Organization report by Scott and Guo [15], inclusion in other published systematic reviews [16–18], or inclusion in the Suicide Prevention Resource Centre Best Practices Registry [19].

The findings from this review will inform clinicians and consumers of the content quality of suicide prevention apps currently available in the marketplace. An examination of the evidence base of app components will assist clinicians in recommending particular apps as part of adjunctive care and those promoting apps through the web to prioritise those most consistent with current evidence. Ultimately, this review will assist consumers to find apps consistent with best practice, and developers to consider the evidence-base of content during app design.

### Objectives

Using descriptive methodology, the primary aim of this study was to compare evidence-based strategies undertaken for suicide prevention with the content of publicly available apps providing tools for suicide prevention.

## Methods

### Eligibility criteria

Huckvale *et al*. highlighted the difference between informational and tool-based apps in a review of apps for asthma [14]. Tool-based apps are “active” or “interactive” as defined by De Jaegere *et al*. [20], specifically requiring active involvement from the user, or allowing users to interact with one another. Meanwhile, “passive” apps are those that solely present content, which could be in a variety of formats such as text or video, but require no user input or interaction beyond navigating through the content. In this review, only “active” or “interactive” apps were included due to the previously identified challenges in identifying the provenance of information contained within suicide prevention apps [21].

In the current review, free and paid-for apps containing content related to suicide were included if they could be downloaded via the official Android and iOS stores. Apps were excluded if they: contained no “active” or “interactive” suicide prevention content; referred to suicide non-literally, for example in branding, or music titles; referred specifically to self-harm with non-suicidal intent; were related exclusively to depression, bipolar disorder, or other mental health conditions, unless suicidality was explicitly mentioned; or were not in English, or included character sets which did not display correctly.

### Information sources

Apps were identified by searching the Australian Google Play store (Android) via its web interface, and the Australian iTunes store (iOS) using its search application programming interface (API). Results were limited by the search engines to a maximum of 250 (Android) or 200 (iOS) apps, and all of these search results were screened.

### Search

A set of core search terms related to suicide was created: suicid*; parasuicid*; kill me/myself/yourself; take my/your [own] life; self[–]harm*; DSH. To ensure consistency in the stemming of search terms across the two app stores, these keywords were manually expanded to create a comprehensive set of terms (see S2 Text). The terms related to deliberate self-harm (DSH) allowed the initial identification of apps where self-harming behaviours are with, without, or with unclear suicidal intention. Search terms on the Google Play store were surrounded by quotation marks to ensure the exact phrases were matched–this functionality was performed automatically by the iTunes search API. The unique identifier, title, description, and price of each app were retrieved from the app store, and apps which appeared in the results for multiple search terms were de-duplicated.

### App selection

During the screening stage, two reviewers independently assessed the title and description of each app against the inclusion and exclusion criteria. The reason for each exclusion was recorded. Results of the screening were compared, and discrepancies were resolved by discussion until consensus was achieved. All apps that were identified as being eligible for inclusion were downloaded and installed on a Samsung Galaxy S4 mini (Android version 4.2) or iPhone 5s (iOS version 7.1) for full content review.

### Data collection process

Following download, each app was opened and assessed independently by the two reviewers to confirm eligibility. The content and features of the apps were then independently reviewed for both harmful and suicide prevention content. The reviewers used a custom coding scheme (see Data Items section), and coded the interventional components directly into a database created for the review. Discrepancies were resolved by discussion until consensus was achieved.

### Data items

Suicide prevention strategies are broad, ranging from population based activities (such as restricting access to means) to specific treatment interventions (such as dialectical behaviour therapy, DBT). Moreover, suicide is commonly experienced in a range of mental and physical health conditions. Apps may also contain potentially harmful content, and may be targeted at different user groups with different purposes (for example consumers, or clinicians). To accommodate these multiple facets, we developed four broad categories on which data were extracted for each app, as described below.

#### App characteristics

The download cost for each app, whether free or paid-for, was recorded. Apps which contained any suicide prevention content were broadly categorised based on their primary function. The following distinct foci were determined: primarily or solely suicide prevention; depression (for example, an app which mentions suicide in the context of depression); deliberate self-harm; physical health or other mental health (for example, an app may include suicide as one of many health topics); or setting-based psychological or general support (for example, a university information app which mentions suicide prevention as part of its welfare programme). This categorisation allowed us to compare apps with a specific focus on suicide prevention and those in which suicide prevention is embedded within a wider context.

#### Harmful content

Harmful information was coded using a synthesis of schemes used by Biddle *et al*. [10], Tam *et al*. [22], and Westerlund *et al*. [23] in their reviews of suicidal content on the internet. The harmful categories were: describing or facilitating access to lethal means; providing encouragement to people to end their life; portraying suicide in a fashionable or appealing manner; or an open category for other harmful content.

#### App quality

In line with previous eHealth and suicide prevention reviews [10, 20, 21, 24] we rated broader app quality indicators. These quality-related features included: the type of developer or provider of the app (Q1); whether the provider name or contact details was explicitly stated within the app (Q2); whether references for the source of app content was included (Q3); whether a privacy policy was included within the app or app store description (Q4); whether the app could be protected with an account login, password or personal identification number (Q5); and whether bugs or reliability issues were apparent through use of the app (although the reviewers did not seek to exhaustively test the app for reliability; Q6).

#### Suicide prevention tools

The spectrum of suicide prevention strategies is wide, spanning public health interventions associated with prevention in the general population, those targeted at higher risk groups, and mental health interventions for treatment and maintenance. In this review, strategies were coded based on the spectrum initially presented by Mrazek and Haggerty [25], and as reported by Scott and Guo in their report for the World Health Organization [15]. For convenience, we divided the strategies into the following five categories: public health, screening, accessing support, mental health/treatment strategies, and follow-up strategies.

#### Public health strategies

Suicide prevention strategies include public health techniques targeting: information about legislation and policies restricting access to lethal means (S1); guidelines for media reporting of suicides (S2); and material about organisational, regional, or national suicide prevention strategies and policies (S3). These public health strategies were expected to be largely information-based and unlikely to be delivered through an interactive mobile app, however they were retained in the coding scheme for completeness.

#### Screening strategies

This category consisted of strategies to improve screening and detection of suicidal risk, with apps targeted at physicians (S4); those in gatekeeper roles (S5); or for individuals to self-screen (S6).

#### Accessing support strategies

Content to encourage or facilitate getting access to help included: requesting help and support from peers or family (S7); accessing help via a gatekeeper (S8); accessing non-crisis support services (S9); and access to crisis support and helplines (S10). For those apps which provided crisis support details, an additional data item was recorded to assess whether the crisis contacts were always visible within the app (for example, through a “get help now” button; S10a), as suggested by De Jaegere *et al*. [20].

#### Mental health/treatment strategies

Mental health strategies focussed on preventing suicide either before or after an attempt. These strategies included: psychotherapy (S11); pharmacotherapy (S12); non-drug physical therapies (S13); the use of safety plans (S14); and postvention support for those bereaved by suicide (S15). As suicide safety plans contain multiple components and address a number of suicide prevention strategies, we performed a separate sub-analysis of the content of these apps. As with the public health category, we did not expect all of these strategies to be deliverable through an app (for example, drug or electroconvulsive therapy), however they were retained for completeness, and to record possible inclusion, for example, as part of a treatment diary.

#### Follow-up strategies

Additional longer-term strategies focussed specifically on follow-up support after a suicide attempt. These strategies included: ongoing outreach and contact (S16); adherence management (S17); and peer support for those who have made a suicide attempt (S18).

#### Evidence quality

After coding the apps’ suicide prevention strategies into the five categories described above, the quality of evidence for each strategy was rated from the extant literature for reducing suicide. As noted previously, we developed a coding scheme based upon the WHO report by Scott and Guo [15], and prevention strategies were coded as having strong evidence (E1) if they were consistent with findings in this report. Supplementary evidence was gathered from reviews by Mann *et al*. [16], Leitner *et al*. [17], and Shekelle *et al*. [18], and strategies with some degree of evidence from these reviews were coded as E2. Finally, if there was a lack of evidence within these reviews, a final check was made with the Suicide Prevention Resource Centre Best Practices Registry [19] to check if the strategy was at least consistent with expert ratings of best practice (E3). Otherwise, we coded a strategy as containing no relevant evidence (E4).

### Summary measures

The number and percentage of apps satisfying each of the coding elements are reported. For each suicide prevention strategy contained within the apps, the coded evidence quality is also reported. The number of strategies and apps containing recognised evidence is reported, along with the number of suicide prevention strategies per app.

## Results

### App selection

**Fig 1:**
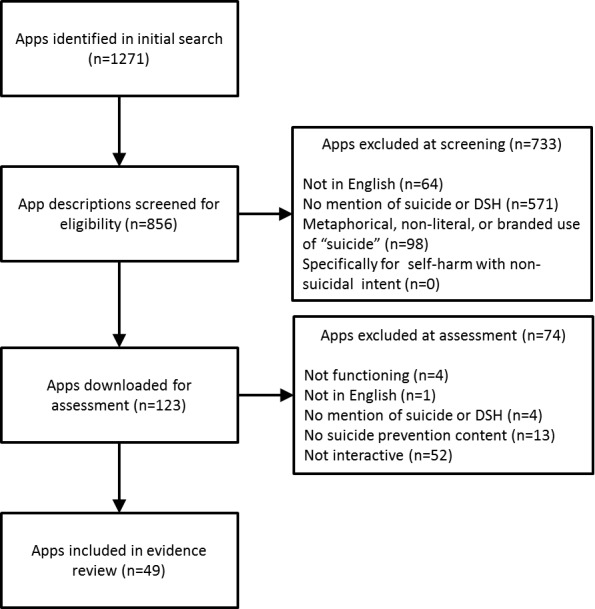
PRISMA flowchart showing the app search, screening, and review.

The PRISMA flowchart for the review is shown in Fig 1. From the original 1271 search results, the descriptions of 856 unique apps were screened, and 123 apps were downloaded for review. Seventy-four apps were excluded following download, including 13 that did not contain any suicide prevention content. Eight of these 13 apps were games with the aim of killing or inflicting harm upon the character, including Russian Roulette. One of the excluded apps suggested risky behaviour, including deliberate self-harm or taking drugs, as an alternative to a suicide attempt. These suggestions contained disclaimers relating to the risk, possible legal consequences, and the lack of concordance with professional medical advice.

Fifty-two of the reviewed apps were excluded as they did not provide interactive features to support suicide prevention. As expected, these informational apps described a wider range of suicide prevention strategies than could be incorporated into a tool-based app. In addition to components identified in the evidence review (described in the following sections), information was provided about: media reporting guidelines (one app; S2), national suicide prevention guidelines (one app; S3), gatekeeper screening for suicide (seven apps; S5), gatekeeper access to support (eight apps; S8), pharmacotherapy (four apps; S12), and non-drug physical therapies (one app; S13). None of these excluded apps contained information on adherence management (S17), or peer-support for those who attempt suicide (S18). The remaining 49 apps (Android: 20, iOS: 29) were included in the evidence review (S1 Dataset).

### App characteristics

All 20 of the reviewed Android apps were free, while seven of the iOS apps required payment to download (four at AU$1.29, one at AU$2.49, one at AU$3.79, and one at AU$18.99). Approximately half of the apps downloaded and reviewed were suicide-specific (n = 24, 49.0%). Of those apps with a wider context, five apps had a focus on depression (10.2%), four on deliberate self-harm (8.2%, none with a specific focus on self-harm with suicidal intent), six on general health information (12.2%), and 10 on general support (20.4%).

#### Harmful content

In addition to the potentially harmful content described for the excluded apps, two additional apps contained a list of means of instant death, although this was presented as suggestions for removing access to means. The risks of presenting lethal means have been discussed in previous work looking at the presentation of suicide on the internet [10, 22].

#### App quality

The majority of apps were developed (Q1) by academic/healthcare institutions, or commercial organisations (20 apps, 40.8%, each). Five apps (10.2%) were privately developed by individuals, and the type of provider was not clear for four apps (8.2%). Despite this range of developers, only 34 (69.4%; 13 suicide-specific apps, 54.2%) included contact details of the provider within the app (Q2).

Although the apps were interactive, delivering a resource to users, just six (12.2%; one suicide-specific app, 4.2%) referenced the source of the content (Q3). While many apps prompted users to enter personal data, less than a half (19 apps, 38.8%; seven suicide-specific apps, 29.2%) included a privacy policy (Q4). Fewer still offered the option to protect the app with an account login, password, or personal identification number (eight apps, 16.3%; three suicide-specific apps, 12.5%; Q5). Nineteen apps (38.8%; eight suicide-specific apps, 33.3%) demonstrated obvious bugs or reliability issues during the content review (Q6).

#### Suicide prevention tools

Table 1 shows the suicide prevention tools that were found in the 49 reviewed apps. The 24 apps which pertained primarily to suicide prevention are shown separately. Accessing peer support and safety plans were the most common features in the apps with a suicide prevention focus, as well as those with a broader focus. Follow-up strategies were least commonly identified within both groups of apps.

**Table 1:** Suicide prevention strategies identified within the reviewed apps.

Prevention strategy	All apps	Suicide-specific apps	Evidence quality
**Public health strategies**			
**S1 Means access restriction**	7 (14.3%)	6 (25.0%)	E3
**Screening**			
**S4 Physician screening**	3 (6.1%)	0	E2
**S6 Self-screening**	13 (26.5%)	2 (8.3%)	E3
**Accessing support**			
**S7 Peer support**	27 (55.1%)	16 (66.7%)	E2
**S9 Non-crisis support**	12 (24.5%)	10 (41.7%)	E2
**S10 Crisis support/helpline**	13 (26.5%)	10 (41.7%)	E1
**S10a Crisis support always visible**	0	0	n/a
**Mental health strategies**			
**S11 Psychotherapy**	2 (4.1%)	0	E2
**S14 Safety plans**	14 (28.6%)	13 (54.2%)	E3
**S15 Postvention**	2 (4.1%)	2 (8.3%)	E2
**Follow-up strategies**			
**S16 Ongoing contact/outreach**	1 (2.0%)	1 (4.2%)	E4
**Total**	49 (100%)	24 (100%)	

Strategies which were not present have been omitted. Values are reported as n (%). Evidence levels are described in the text: E1 (WHO report [15]); E2 (literature reviews [16–18]); E3 (best practice guidelines [19]); E4 (no relevant evidence found).

#### Public health strategies

The only public health strategy identified within the apps related to means access restriction (S1), although this was at the level of an individual, rather than a population. Seven apps (six suicide-specific) allowed the user to identify lethal means that should be removed from their environment in the context of a safety plan (S14). No apps contained interactive features relating to media reporting guidelines (S2), or suicide prevention policies (S3).

#### Screening strategies

Sixteen apps provided interactive screening tools for depression or suicidality: three for mental health professionals (S4), and 13 for individuals to self-screen (S6). No apps provided gatekeeper screening tools (S5).

Two of the self-screening apps were suicide-specific, whereas none of the professional screening apps were primarily focussed on suicide. Nevertheless, one of the professional screening apps contained customised instruments to assist mental health professionals in screening for both suicidality and depression. The second app contained the Hamilton Rating Scale for Depression [26], and the third app included an extended version of the PHQ-9 [27] with additional questions related to suicide, paranoia, hallucinations, and mania.

Of the 13 self-screening apps, two (both suicide-specific) contained a custom screening tool for detecting suicidality, including items on suicidal thoughts, social withdrawal, denying responsibilities, and other warning signs. The remaining self-screening apps focussed on depression: four presented an extended DSM scale, four used a modified or expanded PHQ-9 scale [27], and one which was designed specifically for post-natal depression, reproduced the Edinburgh post-natal depression survey [28]. The remaining two apps provided custom screening tools, one app specifically for depression based on a list of symptoms, and the other app provided multiple custom tools to screen for anxiety, depression, substance use, and suicide.

Eight of the self-screening apps directed users to seek support from health or mental health professionals, or provided crisis support information when users screened high on depression or suicidality measures. Two apps additionally suggested users might be suitable for psychotherapy or antidepressant treatment, but did not directly suggest seeking help. Three further apps did not direct users to help-seeking options, however two were designed to be used as a checklist prior to an appointment with a health professional, and the final app used the screening results to populate a list of suggested tasks, including seeking help, in another section of the app.

#### Accessing support strategies

Apps enabling access to support directed users to either peer support networks, non-crisis support, or crisis support services. None of the reviewed apps provided interactive access to gatekeeper services (S8). Of the apps providing access to help, 27 (16 suicide-specific) apps allowed users to access support from their peers, friends, or family (S7). Approximately half of the apps providing this function did so as part of a safety plan (n = 14; 13 suicide-specific). Eight of the non-safety-plan apps (three suicide-specific) allowed the user to nominate people as supporters and facilitated easy contact during a crisis. The remaining five apps (all non-suicide-specific) additionally allowed users to interact with one another within the app–users could share and discuss common experiences, and support others. This interaction and support took many forms with users interacting through photo sharing in one app, and by video sharing in another. One app also included a personal peer-to-peer support function where users could request support, or nominate times throughout the week when they were available to provide support. Of the five apps which offered peer interaction, four offered some degree of content moderation. Two of these apps specifically indicated that discussion of dangerous, unsafe, or violent acts would be removed; one other app included a function for alerting the service provider about worrying posts; and in one other app all content was centrally approved before being made publicly available.

Twelve apps (10 suicide-specific) also provided access to non-crisis support services (S9), with 11 apps (10 suicide-specific) doing so within a safety plan. The remaining app was developed specifically for a clinical psychology practice and provided active access to the practice via a direct text message.

A further 13 apps (10 suicide-specific) provided access to crisis support services (S10), seven of which (four suicide-specific) were independent of safety plans. Of these, five apps (four suicide-specific) contained interactive crisis support or helpline components. These features included: finding the nearest crisis centre based up location/GPS data (four apps, which were localised versions of the same app); and the ability for the user to enter their own crisis support contact (one app). Two apps also offered features for users to interact with other people. Both apps allowed users to initiate contact with an organisation-affiliated support service, either by text message or online chat capabilities. None of the 13 apps which provided access to crisis support services ensured that this access was visible at all times within the app (S10a).

#### Mental health strategies

Safety planning (S14) was a prevalent mental health strategy contained with the reviewed apps, and is reported separately in a following section. Two apps also provided some degree of interactive psychotherapeutic content (S11), and another two provided postvention support for those bereaved by suicide (S15). No apps provided interactive features related to pharmacotherapy (S12) or physical therapies (S13).

Both of the apps which delivered psychotherapy were based on cognitive therapy–one in the context of depression, and the other for deliberate self-harm. Both apps used thought challenging techniques: the depression app provided a tool that assisted users in identifying and challenging negative thoughts, while the DSH app provided a space to think about negative thoughts and to reframe them positively. The psychological content of both apps was self-guided, with no personalised input from a mental health professional. However, the DSH-orientated app did offer advice based on user-entered responses to motivations behind the current urge to harm, and suggested strategies or activities to distract users until the urge subsided.

The two postvention apps provided an interactive plan for those bereaved by suicide. This was similar to a safety plan, in which the user completed sections of their plan after watching short videos that discussed different aspects of suicide bereavement. Elements of the plan included information, thoughts, and feelings associated with the event, and coping strategies and long term goals. The apps also highlighted that those bereaved by suicide may be at increased risk for suicide themselves, and encouraged seeking support if suicide ideation was present.

#### Follow-up strategies

Finally, one app contained content specifically targeted at supporting someone who survived a suicide attempt. In addition to a safety plan, this app provided an appointment reminder function, which was coded as ongoing contact/outreach (S16). No apps addressed adherence management (S17) or peer support (S18) following a suicide attempt.

#### Safety plans

As discussed above, many suicide prevention tools were incorporated into apps as part of a safety plan (S14). Safety plans were one of the most prevalent app features, with 14 apps (13 suicide-specific) enabling users to create a plan.

Half of these safety plan apps (seven apps, six suicide-specific apps) allowed users to identify lethal means that they should remove from their environment in a crisis (S1). All safety plan apps allowed users to identify peer supporters who could be contacted in a crisis situation (S7). Additionally, 10 of the apps connected to the user’s contact/address book, enabling users to contact peers from within the app (all 10 apps), and facilitating the input of contacts by importing their details from the address book (five apps). In addition to peer support, 11 safety plan apps (ten suicide-specific) included details of non-crisis support services (S9) including psychiatrists, psychologists, mental health organisations and service providers, and general practitioners. All of these apps allowed users to enter their own contacts, and one app additionally assisted users in finding the nearest mental health resource based on location data obtained from the phone handset. Crisis support information (S10) was available within six of the apps (all suicide-specific) and was similar to non-crisis support, allowing users to input relevant crisis support line information. However, one app additionally prompted the user to call a national crisis support centre if pre-nominated warning signs were selected.

Safety plans also contained components not otherwise coded in the description above. Just over half of the safety plan apps (eight apps, seven suicide specific) included a section for users to identify their individual warning signs for a crisis, with two apps (both suicide-specific), also allowing users to actively identify personal triggers. All 14 apps had a section for users to record coping strategies to ameliorate these factors, either allowing the user to enter their own strategies (12 apps), or allowing the user to listen to music or meditation tracks as a means of relaxation (two apps).

In addition to specific means access restriction, an additional six apps featured sections for making the user’s environment generally safer and more comfortable, for example by not being alone. Four apps allowed users to nominate distracting places to go to in a crisis, such as social environments. Finally, seven apps encouraged users to record details associated with medium and longer term life planning, or reasons to live.

### Synthesis of results

Ten distinct suicide prevention strategies were identified within the reviewed apps, one of which was associated with good evidence at level E1 (see **Table 1**). Five strategies were associated with secondary evidence (E2), three with concordance with best practice guidelines (E3), and one for which no relevant evidence could be identified (E4).

Within the 49 apps, 94 individual interactive components were identified. Thirteen components (13.8%) were coded with E1 evidence, 46 (48.9%) were coded as E2, 34 (36.2%) were coded as E3, and one component (1.1%) had no relevant evidence (E4). Sixty individual components were identified in the 24 suicide-specific apps: 10 (16.7%) at E1, 28 (46.7%) at E2, 21 (35.0%) at E3, and one (1.7%) at E4.

Aggregating these results to the app-level, accounting for multiple components within each app and the evidence-based components within safety plans, 13 apps (26.5%; 10 suicide-specific, 41.7%) contained at least one element with some degree of evidence from the WHO report (E1). A further 28 apps (12 suicide-specific) contained at least one component with evidence from the literature reviews (E2), and the remaining eight interactive apps (two suicide-specific) contained at least one element which follows best-practice guidelines (E3). None of the reviewed apps were completely absent of components consistent with evidence or best practice.

Excluding the safety plan apps, a mean of 1.1 (range: 1–2) identified suicide prevention strategies were found in each app. Safety plan apps, which inherently contain multiple components, contained a mean of 3.9 (range: 2–6) components.

Within the 13 apps that contained the one identified high quality strategy coded as E1, the most comprehensive app was a safety plan app [29]. Overall, the most comprehensive app was also a safety plan app [30]. Both these apps were available for Android only. Outwith the safety plan apps, no apps which contained crisis contacts (E1) contained any other coded suicide prevention strategies.

## Discussion

### Summary of evidence

This review has examined app store descriptions for 856 unique apps, the in-app content of 123 apps, and evidence for interactive suicide prevention strategies within 49 apps. Overall, providing access to crisis support services was the only strategy included within apps with E1 level evidence, with approximately a quarter of apps providing this feature. A further half of the apps were consistent with strategies identified in previous evidence reviews, and all apps contained elements consistent with at least best practice guidelines. A small number of apps with potentially harmful content were also identified during the review process.

Twenty-four apps focussing specifically on suicide prevention were identified, all of which included features broadly concordant with the evidence base. This degree of concordance is higher than observed in reviews of other physical and mental health apps, and possibly reflects a higher degree of involvement from professional institutions in app development. For example, Nicholas *et al*. found that only 4% of apps for bipolar disorder were developed by institutions [12], and similarly Shen *et al*. reported that universities and institutions accounted for only 4.2% of developers of depression-related apps [13]. In contrast, institutions accounted for approximately half of developers of the reviewed suicide prevention apps. This potentially accounts for the difference in the proportion of apps that are evidence-informed between the current study and other mental health areas. This may also explain the reasonable provision of duty of care embedded within the relevant suicide prevention strategies. Most apps which offered self-screening tools alerted users towards help seeking options if risk of suicidality was detected, although the suggestion was not always direct or immediate. Apps which allowed users to interact with one another also contained content moderation, which is important considering the potential for sharing potentially harmful content.

Despite the involvement of academic and healthcare institutions in their development, relatively few suicide prevention apps contained broader markers of app quality, such as referencing of source material. Indeed, a review by Aguirre *et al*. [21], specifically of suicide prevention apps, sought to review the evidence base of the content, however found it not possible due to the lack of information within apps indicating the provenance of the content. Apps also suffered from a lack of privacy policies, locking and protecting of apps, and reliability. These deficiencies may influence consumer and professional confidence in these apps.

The components contained within the reviewed apps covered a broad range of suicide prevention content, with the strongest emphasis on safety planning and getting help in a crisis. However, the vast majority of apps only featured one interactive component. Given that the WHO report indicates good evidence for multifaceted suicide prevention strategies, the lack of comprehensive app-based support via the inclusion of numerous tool-based components represents a missed opportunity. Therefore, there is considerable scope for increasing the comprehensiveness of apps for suicide prevention. This could include targeted crisis support for individuals, including immediate access to support services through the app, and an active safety plan (despite the lack of clear evidence for this, it remains best practice and a prudent inclusion). Secondarily, non-crisis tools could include identifying suicide risk factors and triggers, and the delivery of psychological interventions.

In addition to increasing the number of components offered, there is also a need for greater coverage of specific suicide prevention strategies that were missing or under-represented in the reviewed apps. While it may not be feasible to deliver large, public health strategies, pharmacotherapy, or physical therapy through an app, there is room for development of apps to deliver psychotherapy specifically for suicide prevention, improved physician-led screening for suicidality and wider risk factors, and for assertive follow-up following a suicide attempt. Although these strategies lacked the highest grade of evidence, there was some evidence in the literature.

It is perhaps reassuring that there are a number of suicide prevention apps already publicly available to support individuals who may be in crisis, and that the interactive components generally follow best practice guidelines and strategies for which there is at least some degree of evidence. The identification of these good-quality apps, however, remains a challenge. Just under 90% of the apps identified in the app stores contained no suicide prevention strategies, and some contained potentially harmful content. With no regulation in the app marketplace, it currently falls on clinicians and consumers to delineate app quality. Therefore app developers have a challenge in not only creating suicide prevention apps with evidence informed content, but in dissemination strategies so that the app is identified and used by the target audience.

### Limitations

There are a number of possible limitations with the current review. App stores allow publishers to restrict distribution to particular territories, and therefore not all apps may be available globally. As app store searches are localised to one particular country, it is possible that some suicide prevention apps were not found in the search of the Australian stores. However, an ad-hoc search of the term “suicide” on the American, Australian, British, Canadian, French, and German iOS app stores found 100% concordance, and no apps that were not available in each territory. This provides confidence in this review reflecting the global app market.

Unlike searches of literature databases, app store search results provide a static snapshot of a dynamic marketplace. Apps can be updated at any time, removed entirely from the app stores, or disappear from the search results due to decreasing popularity. As an illustration, an ad-hoc search of the Australian iOS store at the time of writing found seven of the 149 apps originally identified through the “suicide” search term were no longer available. This illustrates a methodological challenge inherent in such reviews, as the results can only provide a snapshot into the current offering of available apps. This can also be a challenge for clinicians recommending an app, as there is no guarantee that an app will continue to be available.

Mapping of app components to the evidence base has been, to some degree, hampered by the extent to which both apps and suicide prevention programmes adopt a multifaceted approach. Identifying which features or individual components are effective is therefore a challenge. This is further exacerbated by a relative paucity of good quality evidence for specific suicide prevention interventions that would be appropriate for inclusion into an app. As reported by Leitner *et al*.: “the research literature has adopted a ‘scattergun’ approach… The evidence base for any single form of intervention is therefore very limited” [17]. As there is a lack of a gold standard for effective suicide prevention interventions, we adopted the WHO report as that standard. Implementation of quality suicide prevention strategies, whether in app form or indeed wider policy implementation, could benefit from standard guidelines.

## Conclusions

Despite a lack of evidence in the literature, there are a growing number of apps publicly available for suicide prevention. Many of these provide no interactive features, representing a lost opportunity to engage users in suicide prevention programmes. There are also a small number of apps which, to varying degrees, present potentially harmful content–of greatest concern is the encouragement to engage in risky behaviours such as drugs and deliberate self-harm to manage a crisis. Of those that do provide interactive prevention content, there was limited concordance with high quality evidence-based practice. However, all apps contained at least one component that was broadly consistent with either known evidence or best practice guidelines. While this represents a promising first step in harnessing apps to compliment suicide prevention awareness and strategies, there is a need for suicide-prevention apps to move beyond best practice into the delivery of genuine evidence-based practice with apps supported by empirical data on their effectiveness at reducing suicidal behaviours.

## Supporting Information

S1 Dataset Review Data. (CSV)

S1 Text PRISMA Checklist. (DOC)

S2 Text Full Search Terms. (DOCX)
