# Supplementary material for: A Systematic Assessment of Smartphone Tools for Suicide Prevention
Source: PLoS One. 2016 Apr 13;11(4):e0152285. doi: 10.1371/journal.pone.0152285 (PMC4830444; doi:10.1371/journal.pone.0152285)
Supplement: S2 Text — (DOCX) [file pone.0152285.s003.docx]

# S2 Text. Full Search Terms.

The following is a complete list of terms searched on the app stores:

suicide

suicidal

suicidality

suicidical

suicidism

suicidology

suicided

parasuicide

parasuicidal

self harm

self harmed

self harmer

self harmful

self harmfully

self harmfulness

self harming

self-harm

self-harmed

self-harmer

self-harmful

self-harmfully

self-harmfulness

self-harming

DSH

kill me

kill myself

kill yourself

take my life

take my own life

take your life

take your own life
